# mHealth and wearable technology should replace motor diaries to track motor fluctuations in Parkinson’s disease

**DOI:** 10.1038/s41746-019-0214-x

**Published:** 2020-01-17

**Authors:** M. Kelley Erb, Daniel R. Karlin, Bryan K. Ho, Kevin C. Thomas, Federico Parisi, Gloria P. Vergara-Diaz, Jean-Francois Daneault, Paul W. Wacnik, Hao Zhang, Tairmae Kangarloo, Charmaine Demanuele, Chris R. Brooks, Craig N. Detheridge, Nina Shaafi Kabiri, Jaspreet S. Bhangu, Paolo Bonato

**Affiliations:** 1 Early Clinical Development, Pfizer, Inc, Cambridge, MA USA; 2 Department of Psychiatry, Tufts University School of Medicine, Boston, MA USA; 3 Department of Neurology, Tufts University School of Medicine, Boston, MA USA; 4 Department of Anatomy and Neurobiology, Boston University School of Medicine, Boston, MA USA; 5 Department of Physical Medicine and Rehabilitation, Harvard Medical School, Spaulding Rehabilitation Hospital, Charlestown, MA USA; 6 Wyss Institute for Biologically Inspired Engineering, Harvard University, Boston, MA USA

**Keywords:** Biomarkers, Parkinson's disease, Outcomes research, Drug development

## Abstract

Accurately monitoring motor and non-motor symptoms as well as complications in people with Parkinson’s disease (PD) is a major challenge, both during clinical management and when conducting clinical trials investigating new treatments. A variety of strategies have been relied upon including questionnaires, motor diaries, and the serial administration of structured clinical exams like part III of the MDS-UPDRS. To evaluate the potential use of mobile and wearable technologies in clinical trials of new pharmacotherapies targeting PD symptoms, we carried out a project (project BlueSky) encompassing four clinical studies, in which 60 healthy volunteers (aged 23–69; 33 females) and 95 people with PD (aged 42–80; 37 females; years since diagnosis 1–24 years; Hoehn and Yahr 1–3) participated and were monitored in either a laboratory environment, a simulated apartment, or at home and in the community. In this paper, we investigated (i) the utility and reliability of self-reports for describing motor fluctuations; (ii) the agreement between participants and clinical raters on the presence of motor complications; (iii) the ability of video raters to accurately assess motor symptoms, and (iv) the dynamics of tremor, dyskinesia, and bradykinesia as they evolve over the medication cycle. Future papers will explore methods for estimating symptom severity based on sensor data. We found that 38% of participants who were asked to complete an electronic motor diary at home missed ~25% of total possible entries and otherwise made entries with an average delay of >4 h. During clinical evaluations by PD specialists, self-reports of dyskinesia were marked by ~35% false negatives and 15% false positives. Compared with live evaluation, the video evaluation of part III of the MDS-UPDRS significantly underestimated the subtle features of tremor and extremity bradykinesia, suggesting that these aspects of the disease may be underappreciated during remote assessments. On the other hand, live and video raters agreed on aspects of postural instability and gait. Our results highlight the significant opportunity for objective, high-resolution, continuous monitoring afforded by wearable technology to improve upon the monitoring of PD symptoms.

## Introduction

Parkinson’s disease (PD) is a slowly progressing neurodegenerative disorder with a lifetime risk of ~2% for men and 1.3% for women over the age of 40.^1^ It is the second most common neurodegenerative disorder behind Alzheimer’s disease. While the specific etiology of PD remains unclear, it is generally recognized as a degenerative disease involving the basal ganglia and its projections. The underlying pathology indicates a loss of dopaminergic neurons in the substantia nigra as well as neuronal loss in the locus coeruleus and the raphe nuclei.^2^ The major clinical diagnostic features of PD include bradykinesia, rest tremor, rigidity of skeletal muscles, impairment of postural reflexes, and gait disturbance. Additional symptoms of PD include a large number of non-motor phenomena, including olfactory dysfunction, psychiatric symptoms, sleep disorders, and autonomic dysfunction.^3^

The current clinical management of the disease remains symptomatic with motor symptoms of PD responsive to levodopa treatment early in the disease. Such responsiveness is a key confirmatory diagnostic criterion of idiopathic PD. However, within 3–5 years of beginning treatment with levodopa, ~50% of people with PD begin to experience complications that include motor and non-motor fluctuations, dyskinesia, and psychosis.^3,4^ The pulsatile nature of levodopa delivery provided by orally administered formulations in combination with the progressive denervation of the striatum and effects of PD on other neural structures are likely to blame.^5,6^ With a variety of treatment strategies available and in development to manage complications, reliable and precise monitoring tools are needed both in clinical practice and in clinical trials investigating new treatments.

Questionnaires^7^ and motor diaries^8,9^ remain the primary tools used to identify and monitor motor and non-motor fluctuations. Concerns about the accuracy and reliability of motor diaries are well-appreciated,^10,11^ including the risk of fatigue that may lead to poor adherence by participants, the effects of recall bias,^10^ the limited time resolution they afford, and the nature of the data which measures only the duration of time spent in an identified state and not the severity of impairment (or magnitude of improvement) experienced by individuals with PD in response to treatment.

An alternative to the use of diaries is the serial administration of structured clinical exams, including part III of the Movement Disorder Society Unified Parkinson’s Disease Rating Scale (MDS-UPDRS part III), throughout one or several consecutive medication cycles, as a means to enable a quantitative assessment of the severity of motor fluctuations.^12^ Ideally, this would be done for the duration of a full day. However, the burden to individuals with PD and clinical raters of a full day of live examination involving the same provocative maneuvers repeated many times makes this approach impractical for large scale clinical trials or routine assessments performed in clinical practice.

It may be possible to overcome, at least in part, these limitations by relying on standardized video recordings of the same assessments, for example in the context of a telemedicine visit.^13–15^ However, even when excluding symptoms and maneuvers that cannot be evaluated by video (e.g., evaluations of rigidity or tests of retropulsion), it is not clear to what extent video recordings obscure subtle changes in symptom severity that one can observe in person.

Digital measurement tools including mobile and wearable technologies have been widely recognized as promising for improving upon the remote monitoring of people with PD.^16,17^ The ability of these technologies to make remote, high-resolution, high-frequency observations of ambulation, upper- and lower-extremity movements, and to collect the physiological data (e.g., electromyographic—EMG—and electrocardiographic—ECG data) has been broadly acknowledged.^18–20^

**Fig. 1:**
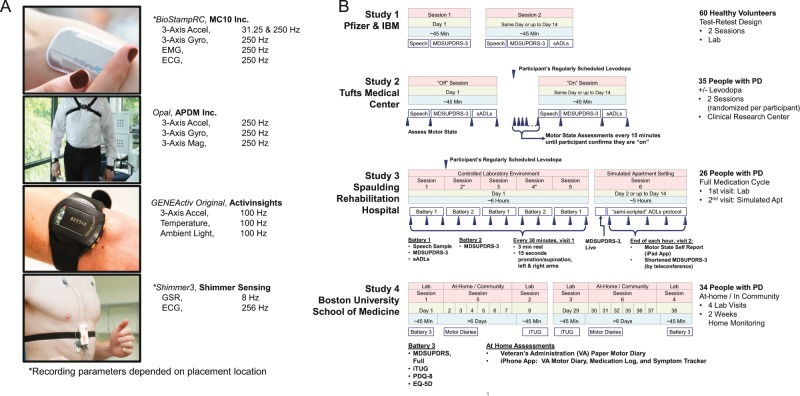
Devices and study designs. **a** Wearable devices and recording parameters that were utilized throughout the project. **b** In studies 1, 2, and 3, multiple administrations of the MDS-UPDRS part III, scripted activities of daily living (sADL’s), and speech tasks were performed. For participants with PD, sessions were timed to take place around PD participants’ regularly scheduled dose of levodopa, and we obtained both a live rating and three video ratings (from separate raters) of the MDS-UPDRS part III for each session. In addition, participants with PD self-reported their motor state multiple times throughout the laboratory sessions. Video raters were blinded to when the participant had taken their medications. In study 4, participants with PD completed 2 weeks of at-home monitoring with the ultimate goal of investigating the relationship between continuous estimates of motor state derived from wearable sensor data, participants’ self-report of their motor state, and the timing of levodopa. BioStampRC image used with permission from MC10. GENEActiv original image used with permission from Activinsights.

To evaluate the potential use of mobile and wearable technologies in clinical trials of new pharmacotherapies targeting PD symptoms, we carried out a project encompassing four studies that investigated potential strategies for monitoring motor and non-motor fluctuations of PD symptoms and complications. We studied changes in motor fluctuators as they followed their own individual levodopa dosing schedule as a model to enable the development of analytical methods for the analysis of sensor data and study their sensitivity to treatment effects on motor states (Fig. 1). In studies 1, 2, and 3, multiple administrations of the MDS-UPDRS part III, scripted activities of daily living (sADL’s), and speech tasks were performed. For participants with PD, sessions were timed to take place around PD participants’ regularly scheduled dose of levodopa, and we obtained both a live rating and three video ratings (from separate raters) of the MDS-UPDRS part III for each session. In addition, participants with PD self-reported their motor state multiple times throughout the laboratory sessions. Video raters were blinded to when the participant had taken their medications. In study 4, participants with PD completed 2 weeks of at-home monitoring with the ultimate goal of investigating the relationship between continuous estimates of motor state derived from wearable sensor data, participants’ self-report of their motor state, and the timing of levodopa intake.

While pitfalls and shortcomings of existing instruments for quantifying motor function and motor fluctuations are often used as a justification for research aimed at developing technology-based tools for PD, several investigators have offered direct evidence. Goetz et al. investigated the agreement between PD patients’ self-assessment of their motor state and blinded video raters in the context a remotely administered UPDRS, and observed only 64% concordance.^21^ Similarly, Stacy et al. observed significant disagreement between patients and clinicans on the presence of wearing-off phenonmena.^7^ In this paper, we further discuss the utility and reliability of self-reports for describing motor fluctuations based on our data set. We also discuss the agreement between participants and clinical raters on the presence of motor complications, and the dynamics of tremor, dyskinesia, and bradykinesia as they evolve over the medication cycle. Other analyses will be the focus of future papers.

## Results

### Demographic and clinical characteristics of the sample

Recruitment for the first study began in July of 2016 and the last subject for the final PD study completed the last visit in April of 2018. For the first study, we recruited healthy volunteers by drawing from the local community in Andover, MA and Yorktown Heights, NY, respectively (Table 1). Sixty healthy volunteers with a mean age of 44 years (23–69 age range) were enrolled, and 33 were female. Compared with PD participants, healthy volunteers were significantly younger (*X*^2^ = 85.18, *p* < 0.000, df = 1), had a higher level of education (*X*^2 ^= 15.22, *p* < 0.00, df = 2) and were more evenly balanced with regard to gender (*X*^2 ^= 4.21, *p* = 0.04, df = 1, Table 1).

**Table 1:** Participant characteristics.

	Healthy volunteers	People with Parkinson’s disease								
	Study #1	Study #2			Study #3			Study #4		
Number of study participants	60	35			26			34		
Age (years)	44.1 ± 10.70 (23–69)	68.3 ± 8.0 (46–79)			66.1 ± 8.2 (42–80)			66.1 ± 8.2 (42–80)		
Height (cm)	171.6 ± 16.4 (67–194)	171.6 ± 16.4 (147–189)			175.4 ± 10.3 (152–196)			171.6 ± 9.8 (155 – 201)		
Weight (lbs)	167 ± 38.0 (101–266)	181.4 ± 41.8 (96–248)			182.7 ± 34.9 (116–260)			167.1 ± 27.5 (120 – 230)		
Ethnicity—male, female, all (N)										
Hispanic	–	0	1	1	0	0	0	0	0	0
Non-Hispanic	–	34	0	34	18	7	25	18	16	34
Unknown	–	0	0	0	1	0	1	0	0	0
Race—male, female, all (N)										
American Indian/Alaska native	–	0	0	0	0	0	0	0	0	0
Asian	–	1	0	1	0	0	0	0	0	0
Native Hawaiian/Pacific Islander	–	0	0	0	0	0	0	0	0	0
Black/African American	–	1	1	2	1	0	1	0	0	0
White/Caucasian	–	19	12	31	16	7	23	18	16	34
Other	–	1	0	1	0	0	0	0	0	0
>1 race	–	0	0	0	2	0	2	0	0	0
Unknown	–	0	0	0	0	0	0	0	0	0
Handedness (%)										
Left	5 (8.3)	6 (17.1)			3 (11.5)			5 (14.7)		
Right	55 (91.7)	29 (82.9)			23 (88.5)			29 (85.3)		
Highest Education (%)										
High school	0 (0)	6 (17.1)			4 (15.4)			4 (11.8)		
College	14 (23.3)	11 (31.4)			13 (50.0)			10 (29.4)		
Postgraduate	46 (76.7)	18 (51.4)			9 (34.6)			20 (58.8)		
First symptom (Yrs)	–	59.5 ± 8.0 (42–75)			53.3 ± 10.6 (31–76)			-		
First diagnosis (Yrs)	–	62.5 ± 8.2 (44–76)			54.8 ± 10.4 (32–76)			–		
MOCA	–	26.0 ± 3.7 (14–30)			27.2 ± 2.5 (21–30)			–		
MMSE	–	–			–			28.8 ± 1.2 (26–30)		
Current medications										
Levodopa (%)	–	35 (100)			26 (100)			34 (100)		
Agonist (%)	–	19 (54)			13 (50)			4 (12)		
MAO-B inhibitor (%)	–	12 (34)			9 (35)			0 (0)		
Anticholinergic (%)	–	2 (6)			1 (4)			0 (0		
COMT inhibitor (%)	–	6 (17)			7 (27)			0 (0		
Hoehn and Yahr (%)										
1	–	2 (6)			0 (0)			8 (24)		
2	–	26 (74)			22 (85)			24 (71)		
3	–	7 (20)			4 (15)			2 (6)		

Ninety-five people with PD were enrolled across the latter 3 studies, and had a mean age of 66 years (42–80 age range), 35 of whom were female. The demographic characteristics of PD participants were virtually identical across studies 2, 3, and 4. While participants recruited to the 2nd study appeared to be slightly older than those recruited to the 3rd and 4th studies (68.3 ± 8.03 years versus 63.3 ± 9.53 and 64.1 ± 6.47, respectively), a Kruskal–Wallis test did not reveal significant differences among these groups (*X*^2 ^= 5.31, *p* = 0.07, df = 2). Likewise, a chi-squared test did not reveal significant differences among the samples recruited according to gender (*X*^2 ^= 2.45, *p* = 0.23, df = 2), or completed education (*X*^2^ = 3.52, *p* = 0.48, df = 4).

### Device data

Over 11,000 h of continuous data were recorded from between 7 and 16 simultaneously worn devices (Supplementary Fig. 1) across the four studies. For recordings made in the context of the laboratory and clinic visits, devices were positioned on the torso, lower back, forearms, wrists, thighs, ankles, and feet, and contained combinations of accelerometers, gyroscopes, magnetometers, barometers, and bio-potential devices recording electrocardiogram (ECG), electromyography (EMG), or galvanic skin response (GSR), depending on the study. In study 4, for recordings made at home, people with PD wore devices on both wrists, the torso, most-affected thigh, and both feet. We utilized devices both with strap-mounted form factors (APDM; ActivInsights) and adhesive-backed patch-worn form factors (MC10), and combination of the two (Shimmer). All in-clinic sessions were monitored with multiple video cameras and microphones to enable off-line assessment of motor status and to collect speech data.

### Diary performance and self-report

In study 4 (NCT03247387), we asked all (*n* = 34) participants to simultaneously complete an electronic (eDiary) version of the Veterans Affairs Patient Motor Diary (VA Patient Motor Diary).^22^ Possible diary entries included: “On”, “On with Troublesome Dyskinesia”, “Off”, and “Asleep”, reported at 30 -min intervals, where participants back-filled “Asleep” entries upon waking each morning. With 3 days of monitoring per week for 2 separate weeks (6 total days of monitoring) and 48 possible entries per day, 288 entries were possible for each participant. The electronic diary allowed us to investigate adherence to eDiary completion by logging each time participants interacted with the application for an initial entry and to subsequently modify an entry.

**Fig. 2:**
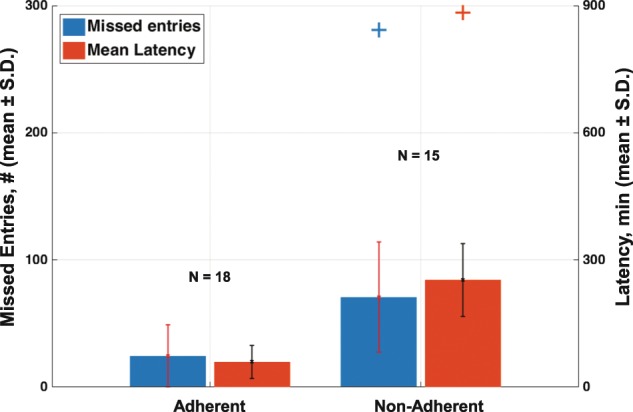
Adherence to motor diaries. All 34 participants in the 4th study were asked to complete an electronic motor diary during the first 3 days of each week of at-home monitoring. One participant’s data were lost due to technical failure of their phone. Among the remaining 33 participants, adherent participants (*n* = 18) rarely missed entries (24.4 ± 24.4 missed) and had low latency to response (59.4 ± 38.6 min), whereas non-adherent participants (n = 15) missed entries more often (70.8 ± 43.5 missed) and took longer to complete those they did not miss (253.0 ± 85.5 min). Data are shown as the mean value, plus, or minus the standard deviation for each measurement.

We observed distinct, varying rates of adherence completing the electronic diary, which we investigated by considering the mean number of missed entries and the mean latency to response (Fig. 2). By visual inspection of the data (Supplementary Fig. 2), we identified distinct adherence patterns: (1) adherent participants (*n* = 18) rarely missed entries (24.4 ± 24.4 missed) and had low latency to response (59.4 ± 38.6 min), (2) non-adherent participants (*n* = 15) missed entries more often (70.8 ± 43.5 missed) and took longer to complete those they did not miss (253.0 ± 85.5 min). Of note, two individuals were particularly non-adherent. We chose to highlight them as they represent unique challenges to researchers employing motor diaries. In one case, a participant missed a similar number of entries to others who were non-adherent (89), but appeared to complete large-numbers of them in a single “batch”, resulting in a high mean latency (884.0 min). Another individual missed the large majority of entries (281 missed) with those few entries completed having a mean latency of 144.2 min. Such late-batch participants and highly non-adherent participants are often the subject of anecdotes by clinical researchers employing motor diaries. In the context of studies utilizing paper versions, it may be possible to avoid contaminating study results by excluding data from non-adherent participants (those who fail to complete the diary) from analysis. However, because it is impossible to know when paper diaries are completed, the results obtained from late-batch completers are indistinguishable from those other participants and may reduce the accuracy of the results.

We further investigated the agreement between participants and clinical raters in studies 2 and 3 throughout the in-lab data collection protocol during each visit. Participants reported their motor state at a variety of time points throughout each session, including prior to and after each administration of the MDS-UPDRS part III. As part of the motor assessment, clinical raters reported whether dyskinesias were present during the exam. In total, 34.8% of the times participants reported being in the “on” state (without dyskinesia), clinical raters indicated that dyskinesias were present. Conversely, in 16.7% of the assessments that participants reported being in the “on with dyskinesia” state, clinical raters indicated that dyskinesias were not present (Table 2).

**Table 2:** Agreement between participants and clinical raters on the presence of dyskinesia.

	Individuals with PD self-reports	
	ON	ON with Dyskinesia
PD specialists presence dyskinesia		
Yes	34.8%	83.3%
No	65.2%	16.7%

Per the instructions of part III of the MDS-UPDRS in studies 2 and 3, clinical raters reported whether dyskinesias were present during each assessment. The study protocol also asked participants to provide self-report of their motor state both before and after the same assessments. We found that 34.8% of the time, participants reported being “on” (without dyskinesia) even though clinical raters indicated that dyskinesias were present during the exam. Conversely, in 16.7% of the assessments that participants reported being in the “on with dyskinesia” state, clinical raters indicated that dyskinesias were not present

### MDS-UPDRS part III scores

Given the above observation that self-reports of motor symptoms may not be reliable and continuous, in-person, visual assessment is not practical, we assessed the agreement of the clinical scores (MDS-UPDRS part III) generated by live raters or raters using video recordings. We pooled scores from live raters and video raters across all participants, all sessions, and all three studies of patients with PD and fit a mixed effects model with relevant demographic, clinical, and experimental variables (see the Statistical methods section for more detail). Because evaluating rigidity requires a physical interaction between examiner and participant and this would not be possible with by remote assessment, we excluded these scores from the analysis. All other subscale scores were included. Significant main effects of Time Since Last Levodopa (*X*^2^ = 24.77, *p* < 0.0001), Hoehn & Yahr stage (*X*^2^ = 40.23, *p* < 0.0001), and Rating Context i.e., live versus video rated, (*X*^2^ = 53.69, *p* < 0.001) were observed. No significant main effects were observed for gender (*X*^2^ = 0.04, *p* = 0.85), years since diagnosis (*X*^2^ = 0.00, *p* = 1.00), or session condition (*X*^2^ = 4.92, *p* = 0.18).

**Fig. 3:**
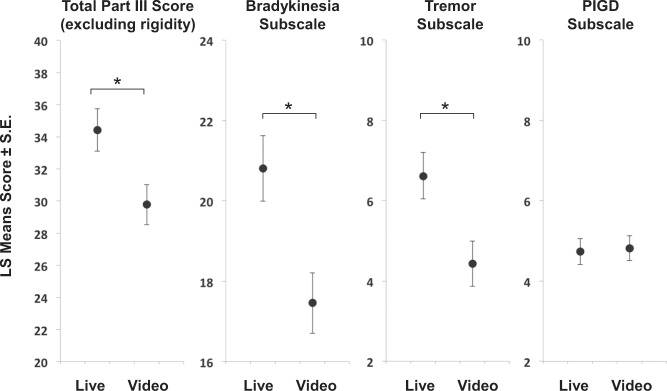
MDS-UPDRS Part III scores, by subscale. The protocol for studies 2, 3, and 4 each included multiple administrations of part III of the MDS-UPDRS. We pooled scores from live raters and video raters across all participants, all sessions, and all three studies of people with PD (*N* = 754 scores) and fit a mixed effects model with relevant demographic, clinical, and experimental variables. When applying this model to the total score (excluding rigidity items), significant main effects of rating context (whether the rating had been performed live or by video) were observed where the video ratings underestimated the score. Post hoc least squares means estimates of the total MDS-UPDRS part III score from video raters was significantly lower than that from live raters by 4.64 ± 0.64 points (left plot, *t* = 7.3, *p* < 0.0001) averaged across session condition and gender. The right three plots show the result of fitting subscale scores for tremor, bradykinesia, and PIGD for the same data set (*N* = 754) using the same mixed model. Post hoc least squares means estimates of the bradykinesia and tremor subscales from video raters were significantly lower than those of live raters (by 3.35 ± 0.45 points, *t* = 7.5, *p* < 0.0001, and 2.18 ± 0.23 points (*t* = 7.5, *p* < 0.0001, respectively). Post hoc least squares means estimates for PIGD from video raters, however, were not significantly different than those from live raters (−0.0878 ± 0.13 points, *t* = −0.675, *p* = 0.5002).

Post hoc least squares means estimates of the Total MDS-UPDRS part III score from video raters was significantly lower than that from live raters by 4.64 ± 0.64 points (*t* = 7.3, *p* < 0.0001) averaged across session condition and gender (Fig. 3). We explored this discrepancy between live and video raters further by fitting linear mixed effects models for component subscales of the MDS-UPDRS part III for bradykinesia (sum of scores from items 3.4, 3.5, 3.6, 3.7, 3.8, 3.14) tremor (sum of scores from items 3.15, 3.16, 3.17, 3.18), and postural instability and gait disorder (PIGD) (sum of scores from items 3.9, 3.10, 3.11, 3.12, 3.13). As with models for the total score, models for tremor and bradykinesia subscale scores revealed significant effects of Rating Context, whereas the model for PIGD did not. These results suggest that while it may be possible to appreciate impairments affecting gait and posture by examining video recordings, subtle differences in the severity of upper- and lower-extremity bradykinesia and tremor are difficult to rate by examining video recordings and prone to underestimation when compared with live ratings.

All raters in each study were MDS-UPDRS certified. The video raters were different individuals than the live raters and were blinded to the medication status of participants. To rule out the possibility that the different ratings provided by live vs. video raters could be attributed to inter-rater differences, we asked live raters to generate clinical scores by inspecting randomly selected video recordings of the same assessments they had previously performed live. This was accomplished ~10 months after the last subject had completed the study. A paired-samples *t* test revealed significantly lower video scores compared with the live ratings (mean of the differences = −2.96, *t* = −2.156, df = 24, *p* = 0.02; Supplementary Fig. 3). This observation indicates that their underestimation of tremor and bradykinesia severity was due to difficulties appreciating subtle movement abnormalities through video.

### Dynamics of motor symptoms

Although different time intervals have been utilized for self-reports of motor states,^11^ motor diaries—including the VA motor diary used in study 4 of this work—often recommend that entries be made every 30 min.^8,9^ Accordingly, we investigated whether collecting data at 30-min intervals is sufficient to capture changes in tremor, dyskinesia, and bradykinesia severity in the participants of study 3. During the laboratory portions of the study, participants performed a battery of scripted tasks that included ADL’s as well as bouts of 15 s of alternating hand movements (Supplementary Table 1). During each of these scripted tasks, performed multiple times throughout the medication cycle, we obtained live clinical ratings of tremor, dyskinesia, and bradykinesia for each extremity.

**Fig. 4:**
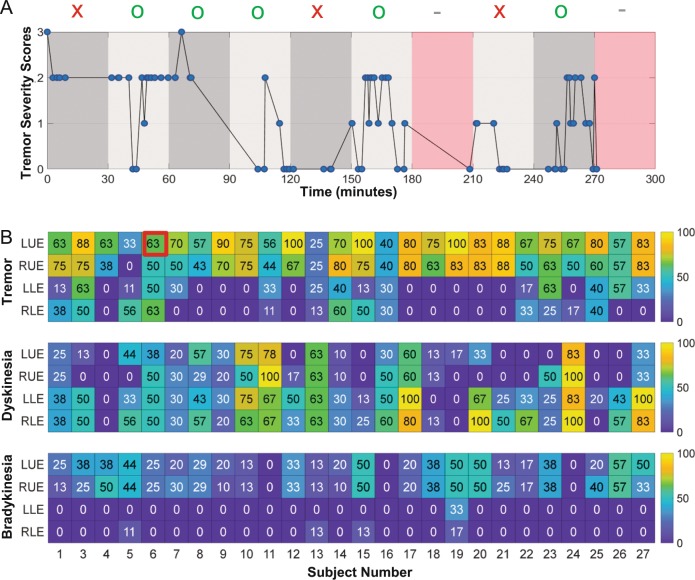
Dynamics of motor symptoms across the medication cycle. **a** Time course of tremor severity from the left upper extremity (LUE) of one participant during the laboratory visit of study 3 (the value of 63% outlined in red in panel **b** was obtained from this time series). Tremor severity was obtained each time a prescribed motor task was performed (whether during one of the several scripted ADLs or the rest, gait and alternating hand movement tasks performed every 30 min). We considered each nonoverlapping 30-min interval during the visit, and excluded any period that did not contain multiple ratings (those periods highlighted red that have a gray dash above them). Among the remaining subset of eight 30-min intervals, we calculated the percentage of periods that contained at least two changes in symptom severity (marked with a green “O” above them). For example, in this participant, rapid fluctuations in symptom severity are apparent in the second, fourth, sixth, and ninth periods. The remaining periods did not contain more than two changes (marked with a red “X” above them). **b** All participants; the percent of 30-min periods during the lab portion of study 3 during which at least two changes to symptom severity occurred. The cell of the matrix representation that is highlighted in red shows the percentage number of instances of at least two changes in tremor severity within a 30-min period derived from the plot shown in panel **a**.

To investigate how frequently changes in the severity of motor symptoms occur (and hence how often data should be collected), we segmented the 6-h visit into nonoverlapping intervals of 30 min for each participant. Figure 4a shows an example of this procedure for subject #6. It highlights the individual 30 -min periods and the severity ratings for tremor of the upper left extremity throughout the study visit. We considered the subset of those intervals where multiple ratings had been obtained (the light and dark gray periods). For that subset of intervals, Fig. 4b shows, for each participant and each body segment (i.e., right and left upper- and lower-extremities), the percentage of intervals in which at least two changes in symptom severity took place. Data are shown for tremor, dyskinesia, and bradykinesia. Across participants, tremor severity changed at least twice in 67% of the 30-min intervals examined for the upper extremities and in 20% of those examined for the lower-extremities. Dyskinesia and bradykinesia scores behaved similar to one another in the upper extremities (in each case, 27% of periods contained multiple fluctuations in severity). In the lower-extremities, however, dyskinesia appeared to fluctuate more often than bradykinesia (44% and 2% of periods examined, respectively).

These results emphasize the significant probability of symptom severity fluctuating at a rate that cannot be captured by using diary entries every 30 min. Importantly, the same consideration applies to live observations of individuals with PD experiencing motor fluctuations. In other words, these results suggest that even live assessments that are carried out at time intervals of 30 min are insufficient to capture the dynamics of tremor, dyskinesia, and bradykinesia, as they evolve over the medication cycle. This observation underlines an important potential advantage of using wearable sensors to track fluctuations in motor symptoms since this approach has potential for generating continuous estimates of the severity of tremor, dyskinesia, and bradykinesia, hence overcoming the limitations of more traditional approaches.

## Discussion

As the search for new pharmacological and non-pharmacological treatments for PD continues, achieving optimal clinical management of motor fluctuations remains a challenging problem.^23^ Significant differences in their pattern and responsiveness to treatment exist across individuals. Thus accurate and reliable monitoring tools that report on treatment outcomes on an individual basis are especially important for this population.

Motor diaries have been extensively utilized to gather treatment outcomes in clinical trials as well as in clinical practice.^8–11^ However, poor adherence has been often reported, particularly in the clinic, where anecdotal evidence of patients completing the diaries immediately prior to their visit is common. The data herein presented show that the use of diaries is marked by a substantial number of missed entries. In fact, ~38% of the participants missed ~25% of total possible entries. Moreover, when non-adherent participants completed the motor diary, they did so with an average delay of >4 h. We also presented evidence that self-reports of the presence/absence of dyskinesia are unreliable. In fact, comparison of self-reports of “on” and “on with dyskinesia” with clinical evaluations by PD specialists showed that self-reports of dyskinesia are marked by ~35% false negatives and 15% false positives. This is not surprising given that individuals with PD often lack an objective perception of their motor status.^24,25^

To address the shortcomings of self-reports, clinicians have investigated serial administration of structured assessments via direct patient observation during a single or multiple motor fluctuation cycles.^12^ Unfortunately, this approach is costly in clinical trials and cumbersome in clinical practice, as well as burdensome to patients. An alternative strategy might be to rely on video recordings and their off-line inspection by PD specialists. This is of interest in the context of a telehealth management of PD^13,14,26^, and can be implemented by instructing patients to videotape themselves at set intervals of times (e.g., every 30 min) during a single or multiple motor fluctuation cycles.^21^ Our results showed that MDS-UPDRS part III scores generated via visual inspection of video recordings (i.e., video rating) of participants with PD experiencing motor fluctuations are marked by a significant bias. In fact, we observed that scores obtained by video rating were lower on average than scores generated by live rating. Interestingly, this was the case for items of the MDS-UPDRS part III related to the assessment of tremor and bradykinesia, but not for items related to the assessment of gait and posture (the PIGD subscale). This result suggests that gross aspects of movement may be accurately assessed from video recordings, whereas more subtle aspects of movement, such as those associated with tremor and bradykinesia, may require live rating.

Would then live assessments performed at intervals of 30 min, although impractical, be the ideal way to assess motor fluctuations in PD? Our results suggest that this approach would not accurately capture the dynamics of fluctuations in the severity of tremor, dyskinesia, and bradykinesia. In fact, we showed that ~ 67% of the 30-min intervals examined for the upper extremities contained at least two changes in tremor severity, ~27% of such intervals showed at least two changes in bradykinesia severity, and ~44% of the 30-min intervals examined for the lower-extremities showed at least two changes in dyskinesia severity.

Mobile and wearable technologies provide a means to generate estimates of PD symptoms with high time resolution.^16^ They may also offer better sensitivity that human observers may be capable of enabling the detection of subtle changes previously impossible to detect. A large body of literature shows that algorithms designed to analyze the data collected during the performance of scripted motor tasks provide accurate estimates, compared with clinician ratings, of the severity of PD symptoms.^27–34^ However, future work is needed to derive accurate estimates of the severity of PD symptoms from the data collected during the performance of unscripted activities, namely during the performance of ADL’s.^17,35^ One possible approach suitable to analyzing data collected during the performance of unscripted activities consists of implementing the cascade of two modules. The first would be devoted to selecting data segments suitable for detecting and assessing the severity of PD symptoms. The second module would be devoted to generating estimates of symptom burden and severity. For example, rest tremor assessment might proceed with the first module identifying segments of time when a body segment is not undergoing voluntary motion, and the second module generating estimates of the severity of tremor at rest. Similarly, to assess gait impairments using the second module, episodes of gait would be first identified with the first module.

With the approach proposed above, data collected using inertial sensors in the home and community settings could provide estimates of the severity and fluctuations of tremor, dyskinesia, and bradykinesia, three of the four cardinal features of PD. However, the fourth feature, rigidity, remains difficult to monitor by inertial sensors alone, because current assessments of rigidity require the physical interaction between a trained expert and the individual under examination. To overcome this issue and explore the possibility of measuring rigidity during voluntary motion, in each of the studies, we collected EMG and speech data with the intention of investigating relationships between their characteristics and rigidity.^35^ Speech is affected by PD,^36^ and speech characteristics can be related to UPDRS scores,^37^ but an association with fluctuations in rigidity severity has not yet been established. Ongoing analyses of the data collected in the current project will determine if speech characteristics can be utilized as a proxy for the severity of rigidity.

Another important issue that requires future work is the development of methods to assess fluctuations in non-motor symptoms in the home and community settings. The relevance of achieving optimal clinical management of non-motor fluctuations cannot be overstated.^23,38,39^ Encouraging results have been obtained by others suggesting that certain parameters describing heart rate variability are strongly associated with the severity of autonomic dysfunction in PD.^40–44^ However, it remains to be determined if continuous monitoring of heart rate variability will reveal fluctuations in non-motor symptoms. Ongoing analyses of the ECG and GSR data collected in this project will allow us to explore the potential role of these measures in monitoring fluctuations in non-motor symptoms of PD.

These considerations highlight important areas of future and ongoing work, and the results summarized in this manuscript show the opportunity to improve PD monitoring, for example by using mobile and wearable technologies to assess fluctuations in motor symptoms of PD and specifically fluctuations in the severity of tremor, dyskinesia, and bradykinesia. The fast dynamics of these phenomena requires that estimates of their severity be generated with high time resolution, which makes impractical not only the use motor diaries but also to rely on the serial performance of structured assessments. Hence, mobile and wearable technologies should be utilized to complement (if not replace) traditional approaches—such as motor diaries—to assess fluctuations in motor symptoms. However, self-reports appear to still be of great relevance to capture fluctuations in non-motor symptoms until associations between fluctuations in non-motor symptoms and physiological data (e.g., heart rate variability) are established.

## Methods

### Statistical methods

To investigate the similarity of our healthy control and PD cohorts, we performed chi-squared tests of independence to evaluate the relationship between demographic variables (age, gender, and completed education) and cohort membership (healthy control and PD). Likewise, to investigate the homogeneity of our sample of PD participants across studies 2, 3, and 4, we performed the same chi-squared test to evaluate the independence of relevant variables (age, gender, completed education, and Hoehn and Yahr stage) from studies 2, 3, or 4.

In studies 2, 3, and 4, the administration of part III of the MDS-UPDRS was timed, per protocol, to take place either immediately prior to the participants’ next dose of levodopa (labeled as an “off” condition), after a dose and self-reported confirmation of the participant feeling “on” (the “on” condition), or at time points in between (labeled as “transitioning to off” or “transitioning to on” conditions). In studies 2 and 3, we obtained live in-person ratings as well as 3 ratings from video of symptom severity. These scores were pooled with scores from study 4 and used to build a linear mixed model that fit the MDS-UPDRS part III total scores with Session Condition (On, Off, Transitioning to On, or Transitioning to Off), Rating Context (Live vs. Video Recorded), Years Since Diagnosis, Gender, Hoehn & Yahr Stage, and Time Since Last Levodopa (min) as fixed effects, and Subject as a random effect. Significant effects (ANOVA findings) were followed with post hoc comparisons for least squares means across factor levels.

### Recruitment and eligibility

The project encompasses four observational studies, including both healthy volunteers and people with PD. Healthy volunteers were recruited if they were between the ages of 18 and 70. Potential participants were excluded if they reported the existence of an implanted medical device, such as a pacemaker or implanted pump, were pregnant or had any condition that would prevent them from completing study activities. Participant self-report was used to confirm eligibility.

Eligibility criteria for people with PD were intended to be representative of criteria for ongoing clinical trials investigating therapeutic interventions aimed at reducing motor fluctuations. People with PD were recruited to studies 2, 3, and 4, if they had a current clinical diagnosis of PD consistent with the UK Parkinson’s Disease Society Brain Bank Clinical Diagnostic Criteria.^45^ Studies 2 and 3 required evidence from a qualified neurologist, while study 4 required self-report of diagnosis. All participants had a Hoehn and Yahr stage less than or equal to 3, when assessed in the “on” state. They were required to be responding to levodopa, and be on a stable dose for at least 4 weeks prior to their first assessment. They had to confirm their ability (self-report) to recognize their “wearing-off” symptoms and confirm that they improved after the next dose of PD medication.

People with PD were excluded if they had any current history of neurological disease (other than PD), cognitive impairment, or psychiatric illness that in the investigator’s judgment would interfere with subject participation. They were also excluded if they currently had cardiac pacemakers, electronic pumps, or any other implanted medical devices, including deep brain stimulation devices.

### Study 1: healthy volunteers

The first study was a non-interventional study conducted in 60 healthy volunteers at two different sites (41 volunteers in MA, 19 in NY) in order to develop an acceptable testing protocol and explore the reliability of outcomes captured by devices in a controlled laboratory setting. Written informed consent was obtained from all participants, all relevant ethical regulations were complied with, and the protocol was approved by Schulman’s commercial Institutional Review Board. The protocol was designed with two sessions each lasting approximately an hour conducted either during a single visit the same day or during two visits on different days up to 14 days apart. A portion of the protocol has been described previously.^46^

All assessments were conducted in a controlled laboratory environment. Participants donned 14 wearable devices from two manufacturers (APDM and MC10; Supplementary Fig. 1). Recordings were initiated at the beginning of each session and ran continuously throughout the session.

Both visits began with the performance of speech and motor tasks associated with administration of part III of the MDS-UPDRS. A study staff member facilitated the performance of activities and recorded the timing of the beginning and ending of each using an electronic CRF application on an iPad. In addition, several balance and mobility assessments (Mobility Lab, APDM, Inc.) were performed including: (i) 5-times sit-to-stand, (ii) 360° turn, (iii) postural sway (30 s eyes open and 30 s eyes closed conditions), and (iv) 2 min stand-and-walk. During the second visit, in addition to part III of the MDS-UPDRS and Mobility Lab assessments, participants completed a series of scripted ADLs representing fine motor tasks, dressing behaviors, eating behaviors, and balance and mobility-related behaviors. These tasks were subsequently and identically administered to participants in studies 2 and 3. The instructions given to the participant during these scripted ADLs are provided in Supplementary Table 1. At the end of the study, participants completed a questionnaire aimed at assessing the overall comfort of the devices, whether any device or placement location was particularly uncomfortable, and how likely they would be to wear either device type continuously at home for multiple days.

### Study 2: people with PD before and after levodopa

The second study was a non-interventional study conducted at a single site in 35 people with mild-to-moderate PD under controlled laboratory conditions. Participants were asked to complete a similar protocol as was done in the first study. Data collection was carried out at the clinical and translational research center (CTRC) at Tufts Medical Center. Written informed consent was obtained from all participants, all relevant ethical regulations were complied with, and the protocol was approved by the Tufts Health Sciences Campus Institutional Review Board. Data collection was carried out during two sessions, either during a single visit on the same day or over two visits on separate days, up to 14 days apart. All participants completed one of the sessions immediately prior to a regularly scheduled dose of levodopa (“off” condition), and the other session after they took their medication and reported feeling “on” (the “on” condition). The order of the two sessions was randomized across participants.

Upon arriving at the clinic for their first session, participants completed a study questionnaire that included the 9-item wearing-off questionnaire (WOQ-9). Next, during both sessions, an MDS-certified movement disorders neurologist administered part III of the MDS-UPDRS. During both sessions, all participants completed the same APDM Mobility lab and scripted ADL battery as was performed by healthy volunteers in study #1.

### Study 3: people with PD throughout a complete levodopa medication cycle

The third study was a non-interventional study in 26 people with mild-to-moderate PD (26 enrolled, 25 completed both visits 1 and 2). Participants completed two visits during which they donned the same sensors as in studies 1 and 2, and additionally shimmer sensors to record GSR and ECG. Written informed consent was obtained from all participants, all relevant ethical regulations were complied with, and the protocol was approved by the Spaulding Rehabilitation Hospital Institutional Review Board. On the morning of each visit, participants took their normal morning dose of medication and reported to the site 1 h prior to their second normally scheduled medication dose for a visit that lasted approximately the duration of one levodopa medication cycle (~6 h). Continuous recordings were initiated and continued for the duration of the visit.

During the first visit, which took place in a controlled laboratory, participants performed multiple (up to 5) repetitions of the same battery of tasks described for the previous two studies that included a speech assessment, part III of the MDS-UPDRS, and a series of scripted ADLs and mobility tasks. The first repetition was timed to occur just before taking their regularly scheduled medication. Next, participants began self-reporting, at 15-min intervals, whether they felt “on.” During this transitioning period, if time allowed, a second battery was performed (a “transitioning to on” session). Once they reported feeling “on” they completed a third battery (an “on” session). The battery of tasks, which lasted ~30 min, was then repeated two more times (“transitioning to off” sessions) at 1-h intervals with the last repetition timed just before their next scheduled dose of medication.

The second visit took place in a simulated apartment-style living environment. Participants were asked to perform activities of daily living that were self-selected from a different list for each hour they stayed in the apartment (Supplementary Table 2). During this visit, they wore the same devices and had the option of being accompanied by a partner or caregiver. The list was provided to them at the beginning of the visit. Some of the items were repeated each hour (assembling nuts and bolts and visiting every room twice), while others were different. In addition, each hour throughout the visit, study staff members connected to the participant through video conferencing software and administered a shortened motor assessment based on a subset of MDS-UPDRS part III tasks.

### Study 4: people with PD at home and in the community

The fourth study (NCT03247387) was a non-interventional study in 34 people with PD. The primary objective was to investigate the agreement between paper and electronic versions of the VA patient motor diary. Secondary and exploratory objectives included investigating the relationship between continuous measures of motor function made by wearable sensors, participant self-report of their motor state, and the timing of Levodopa intake. The study was performed at Boston University School of Medicine. Written informed consent was obtained from all participants, all relevant ethical regulations were complied with, and the protocol was approved by Boston University School of Medicine Institutional Review Board. People with PD and currently responding to Levodopa participated in two one-week periods of continuous monitoring with wearable devices at home. Both periods began and ended with laboratory visits (a total of 4) and were separated by ~1 month. During the first and last visits (approximately days 1 and 38), the full MDS-UPDRS was administered by an MDS-certified neurologist. In addition, during all four lab visits, participants completed paper versions of the Parkinson’s Disease Questionnaire, 8-point (PDQ-8), and EuroQOL 5-Dimension (EQ-5D)) as well as a 7-meter timed-up-and-go (TUG) while wearing sensors. Paper and electronic motor diaries were completed for 3 consecutive days during each of the week-long at-home monitoring periods. The mobile application enabling the electronic motor diary also included a medication questionnaire, the EQ-5D, PDQ-8, and collected timestamps of data entry enabling monitoring of several adherence metrics, including whether an entry was missed, a value was later modified, and the latency between data entry and the prescribed diary period.

### Reporting summary

Further information on research design is available in the Nature Research Reporting Summary linked to this article.

## Supplementary information

Supplementary Information

Reporting Summary

## Data Availability

The data that support the results reported herein can be obtained upon request of the four institutions that were the legal sponsors of the respective studies subject to the terms of informed consent provided by participants.

## Code availability

To collect at-home self-reports of motor state in study #4 and monitor adherence metrics, we relied on a custom iPhone application built specifically for the study. All other data for study #4 reported herein (demographics, and clinical scores) were collected manually without the use of software code. Data analysis was carried out using Matlab (for the dynamics of motor symptoms) and R (to compute descriptive statistics for demographic variables, implement the mixed model for investigating MDS-UPDRS Part III scores, and perform hypothesis testing of demographic variables). The code used to complete the analysis may be available upon request.
