# Supplementary material for: mHealth and wearable technology should replace motor diaries to track motor fluctuations in Parkinson’s disease
Source: NPJ Digit Med. 2020 Jan 17;3:6. doi: 10.1038/s41746-019-0214-x (PMC6969057; doi:10.1038/s41746-019-0214-x)
Supplement: Supplementary file 1 — Supplementary Information [file 41746_2019_214_MOESM1_ESM.pdf]

Supplementary Figure 1
Device placement and recording parameters, all studies.

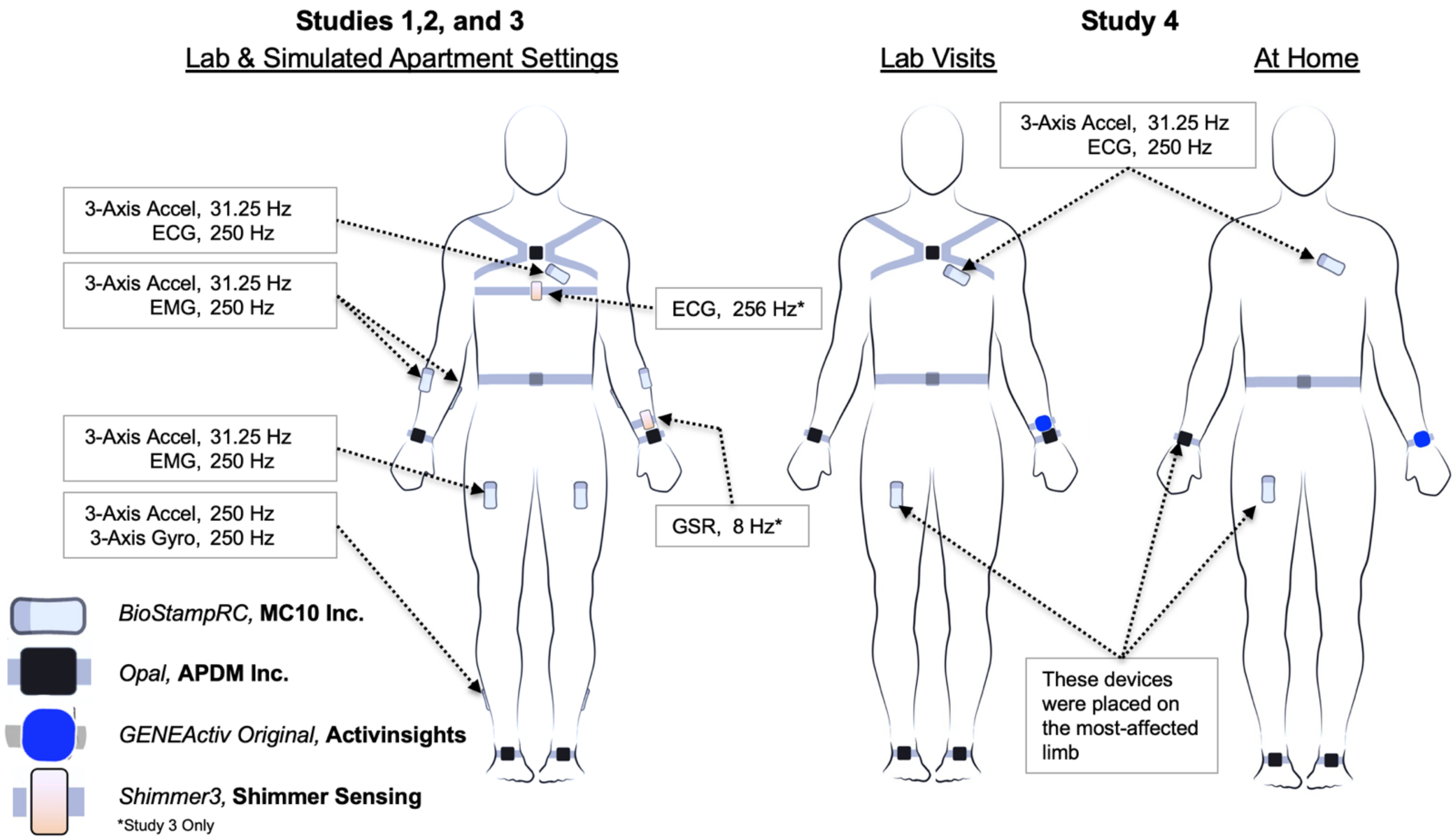

Supplementary Figure 2
Adherence to motor diaries at home

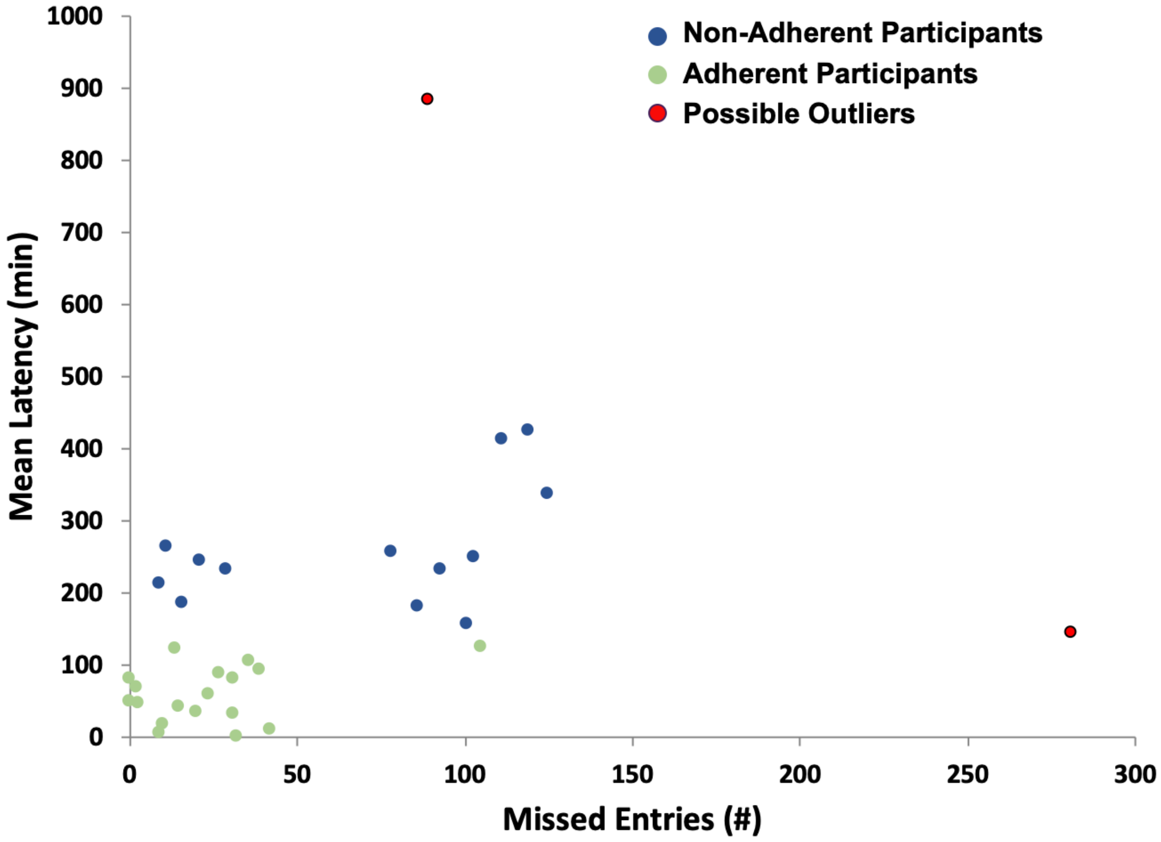

In study 4, electronic motor diaries were completed during the first three consecutive days during each of two weeks of at home monitoring. At 30-minute intervals over 6 full days of monitoring, 288 total entries were expected from each participant. The electronic diary monitored the timing of data entry and recorded, whether an entry was missed or a value was later modified, allowing for a quantitative assessment of participant adherence as measured by the number of missed entries and entry latency. The plot shows summary metrics for each of 33 participants.

Supplementary Figure 3
Differences between video and live ratings of symptom severity as measured using the MDS-UPDRS part III

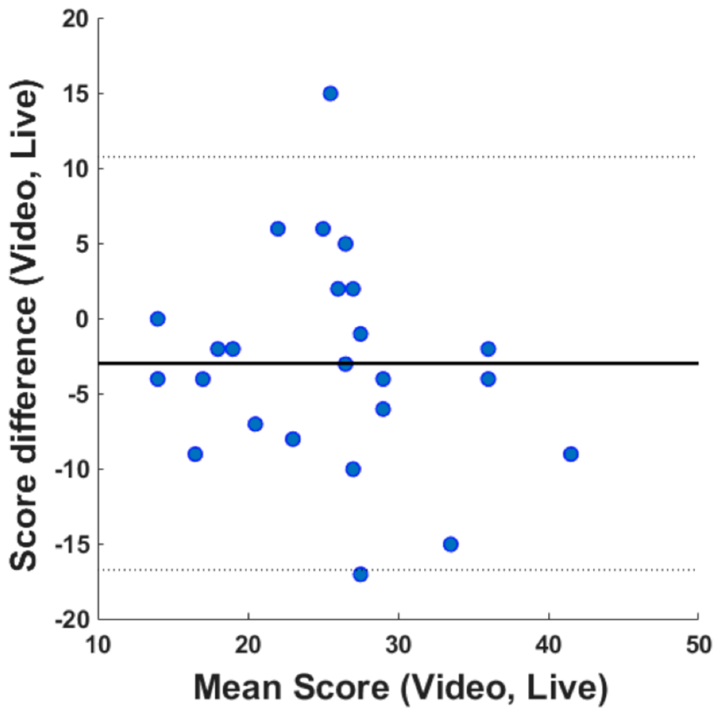

For the analysis shown in Figure 3, video raters were different individuals than live raters and blinded to medication status. To rule out the possibility that differences between live vs. video raters could be attributed to inter-rater differences, we asked live raters to generate clinical scores by inspecting 25 randomly selected video recordings of the same assessments they had previously performed live more than 10 months prior. Each point in the figure is one assessment.

Supplementary Table 1: Scripted Activities of Daily Living

Item	Activity	Category
1	Write the following sentence: "There was an old woman who lived in a shoe"	Fine Motor Tasks
2	Write the sequence of letters "elelelel" in cursive 10 times, down the page	
3	Fold a piece of paper in half 4 times	
4	Enter three sequences of numbers (507,169,746) into a remote control	
5	Tie then untie both shoes	Dressing Behaviors
6	Put on a lanyard and bracelet, then remove both	
7	Approach a coat rack; take a lab coat off the hook and put it on; button all the buttons then unbutton all buttons; remove the coat and return it to the rack	
8	Approach a coat rack; take a sweatshirt off the hook and put it on; zip and then unzip the sweatshirt; remove it and return it to the rack	
9	Shake a bottle of water 5x, then open the cap and take a sip	Eating Behaviors
10	Pour a cup of water, drink, return to table, and drink again	
11	Pour water into a bowl and take two spoon fulls like eating soup	
12	Approach a closet door, open it, look in, then close	Balance and Mobility-Related Behaviors
13	walk out and back 10 m carrying a book and place it on a table	
14	walk out and back 10 m carrying a heavier item (bag with >10 lbs)	
15	Stand holding the heavier bag for 90 seconds	

Supplementary Table 2: Non-Scripted Activities of Daily Living

Item	Activity Instructions	Hour 1	Hour 2	Hour 3	Hour 4	Hour 5
1	Make sure to visit every room in the apartment at least twice.	✓	✓	✓	✓	✓
2	Find the box labeled, "Motor Control Tasks" found in the kitchen. You will find a set of directions in the box that describes each activity. Pick any one activity not done in a previous session and perform for a total minimum of 5 minutes.	✓	✓	✓	✓	✓
3	Find the nuts and bolts in the box. Put together and take apart 5 nuts and bolts.	✓	✓	✓	✓	✓
4	Find the grocery bags in front of the door and take them to the kitchen. Unload the groceries and place them in the refrigerator or upper cabinet.	✓				
5	Take the dustpan and broom, located in the back corner of the kitchen. Sweep up the piles of dirt in the kitchen and hallway leading up to the bedroom. Throw dirt out in the trash can.	✓				
6	Prepare and eat a snack and beverage from the list of options.	✓				
7	Get in and out of bed twice while leaving your shoes on.		✓			
8	Make the bed.		✓			
9	Find the shirts and pants located in the bedroom. Fold them then place them in the box. Close the box and place it back where you found it.		✓			
10	Brush teeth in the bathroom using disposable tooth brush and toothpaste located on the sink.		✓			
11	Prepare a meal from the menu options provided. Do not eat the meal until hour 4.			✓		
12	Set the table with the: table cloth, plates, utensils, cups, beverage of your choice.				✓	
13	Eat the meal prepared in hour 3.				✓	
14	Load the dishwasher					✓
15	Clear the table					✓
16	Wipe the countertop and table using cleaning solution and paper towels					✓
